# Treatment of massive hemoptysis after thoracic aortic aneurysm repair

**DOI:** 10.1186/s42155-022-00293-3

**Published:** 2022-03-15

**Authors:** Fumio Morimura, Kohei Hamamoto, Hiromi Edo, Osamu Ishida, Koji Tsustsumi, Soichiro Tamada, Hiroshi Kuwamura, Yasuhiro Enjoji, Yohsuke Suyama, Hiroaki Sugiura, Sadahiro Watanabe, Ippei Ozaki, Hiroshi Shinmoto

**Affiliations:** 1 Department of Radiology, National Defense Medical College, 3-2 Namiki, 359-8513 Tokorozawa, Saitama Japan; 2 Department of Cardiovascular Surgery, National Defense Medical College, 3-2 Namiki, 359-8513 Tokorozawa, Saitama Japan; 3 Department of Radiology, Japan Self-Defense Forces Central Hospital, 1-2-24, Ikejiri, Setagaya-ku, 154-0001 Tokyo, Japan

**Keywords:** Hemoptysis, Transcatheter arterial embolization, Thoracic endovascular aortic repair, Pulmonary ligament artery

## Abstract

**Background:**

Massive hemoptysis after thoracic aortic aneurysm (TAA) repair is a rare but potentially lethal condition. Endovascular management is a challenging treatment option due to the complexity of culprit vessel access.

**Case presentation:**

An 81-year-old woman was referred to our hospital with massive hemoptysis. She had a history of graft replacement and thoracic endovascular aortic repair (TEVAR) for dissecting TAA. Computed tomography (CT) showed massive atelectasis with hematoma in the left lower lung lobe adjacent to the descending aortic aneurysm treated with TEVAR. Contrast-enhanced CT revealed a pseudoaneurysm and proliferation of abnormal vessels at the peripheral side of the left pulmonary ligament artery (PLA) in the atelectasis. The PLA continued to the right subscapular artery via a complex collateral pathway. Diagnostic angiography of the right subcapsular artery revealed a pseudoaneurysm and abnormal vessels at the peripheral side of the left PLA with a systemic-pulmonary artery shunt. Transcatheter arterial embolization (TAE) for the left PLA via the collateral pathway with *N*-butyl cyanoacrylate achieved complete embolization. The patient’s hemoptysis was controlled and she was discharged.

**Conclusions:**

Here we presented a case of massive hemoptysis due to PLA disruption that occurred after TAA repair. TAE via a complex collateral pathway is a feasible and effective treatment for hemoptysis, even in patients who have undergone surgical or endovascular TAA repair.

## Background

Massive hemoptysis is a potentially lethal condition that commonly occurs as a complication of bronchiectasis, pulmonary tuberculosis, lung cancer, and pulmonary aspergillosis (Chun et al. 2010a, b; Julià-Serdà et al. 1996a, b). It also occurs as a complication of ruptured or repaired thoracic aortic aneurysm (TAA) and most often of aorto-bronchial fistula (ABF) (Czerny et al. 2015a, b). Hemoptysis other than ABF commonly originates from the bronchial artery, but non-bronchial intercostal, internal thoracic, and pulmonary ligament arteries (PLA) can also be involved (Chun et al. 2010a, b; Julià-Serdà et al. 1996a, b; Liu et al. 2013a, b). Transcatheter arterial embolization (TAE) is considered an effective and less invasive treatment for hemoptysis (Chun et al. 2010a, b). However, this method is challenging in cases after TAA repair because of the complexity of the access to the responsible vessel.

Here we report a case of massive hemoptysis due to PLA disruption that occurred after surgical and endovascular TAA repair that was successfully treated with TAE via a complex collateral pathway.

## Case presentation

**Fig. 1:**
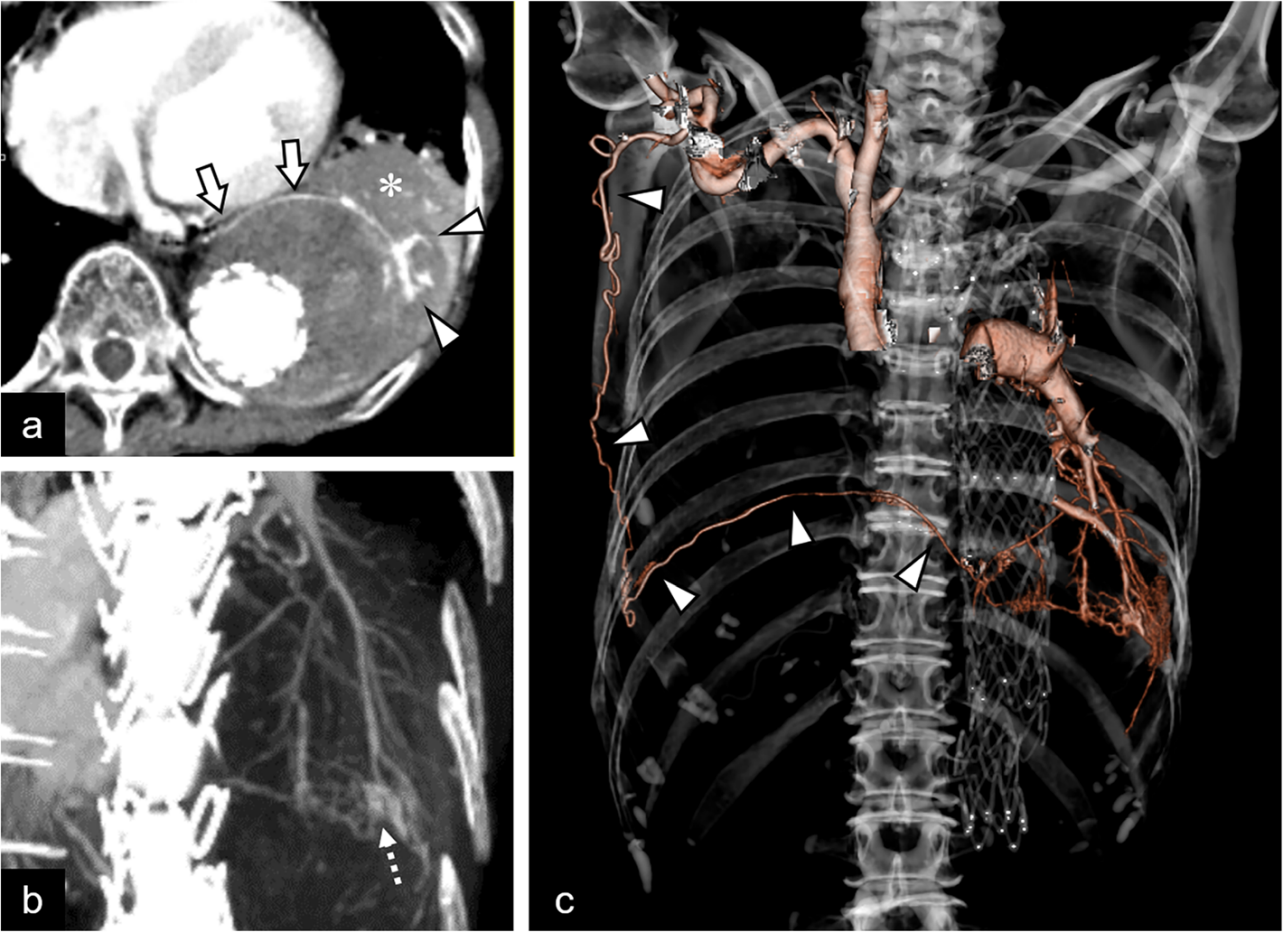
Computed tomography (CT) findings. **A** Contrast-enhanced CT showing the large atelectasis (asterisk) adjacent to a thoracic aortic aneurysm treated with a stent graft. The arrows and arrowheads indicate the hypertrophied left pulmonary ligament artery (PLA) and abnormal proliferative vessels in the atelectasis, respectively. **B** Slab-maximum intensity projection image in the oblique coronal view. Abnormal proliferation of small vessels is observed at the peripheral side of the left PLA. The dashed arrow indicates a pseudoaneurysm. **C** Volume-rendering image. The continuity from the right subscapular artery to the left PLA via the tortuous complex collateral pathway is clearly visible (arrowheads)

**Fig. 2:**
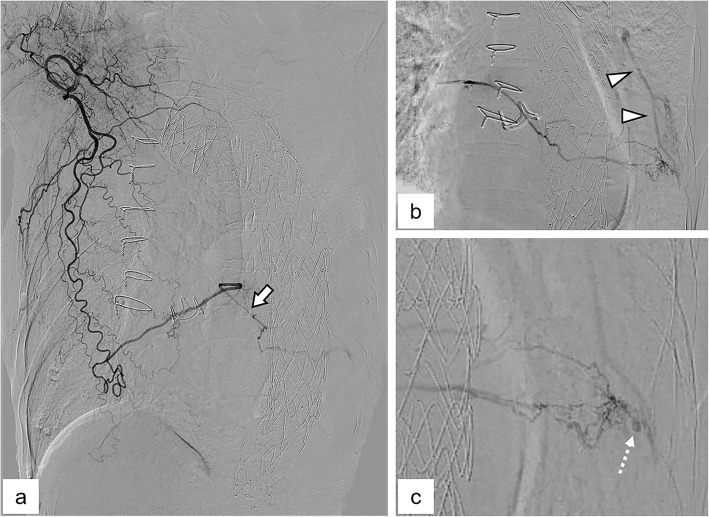
Angiographic findings. **A** Selective digital subtraction angiography (DSA) of the right subclavian artery. Consistent with contrast-enhanced computed tomography image, the continuity from the right subscapular artery to the left pulmonary ligament artery (PLA) (arrow) is shown. **B** and **C** Selective DSA of the left PLA. Note that the abnormal proliferation of small vessels is visible at the peripheral side of the left PLA. The arrowheads in b indicate the reversal blood flow of pulmonary artery, suggesting the presence of a systemic-pulmonary artery shunt. The dashed arrow in c indicates the pseudoaneurysm

**Fig. 3:**
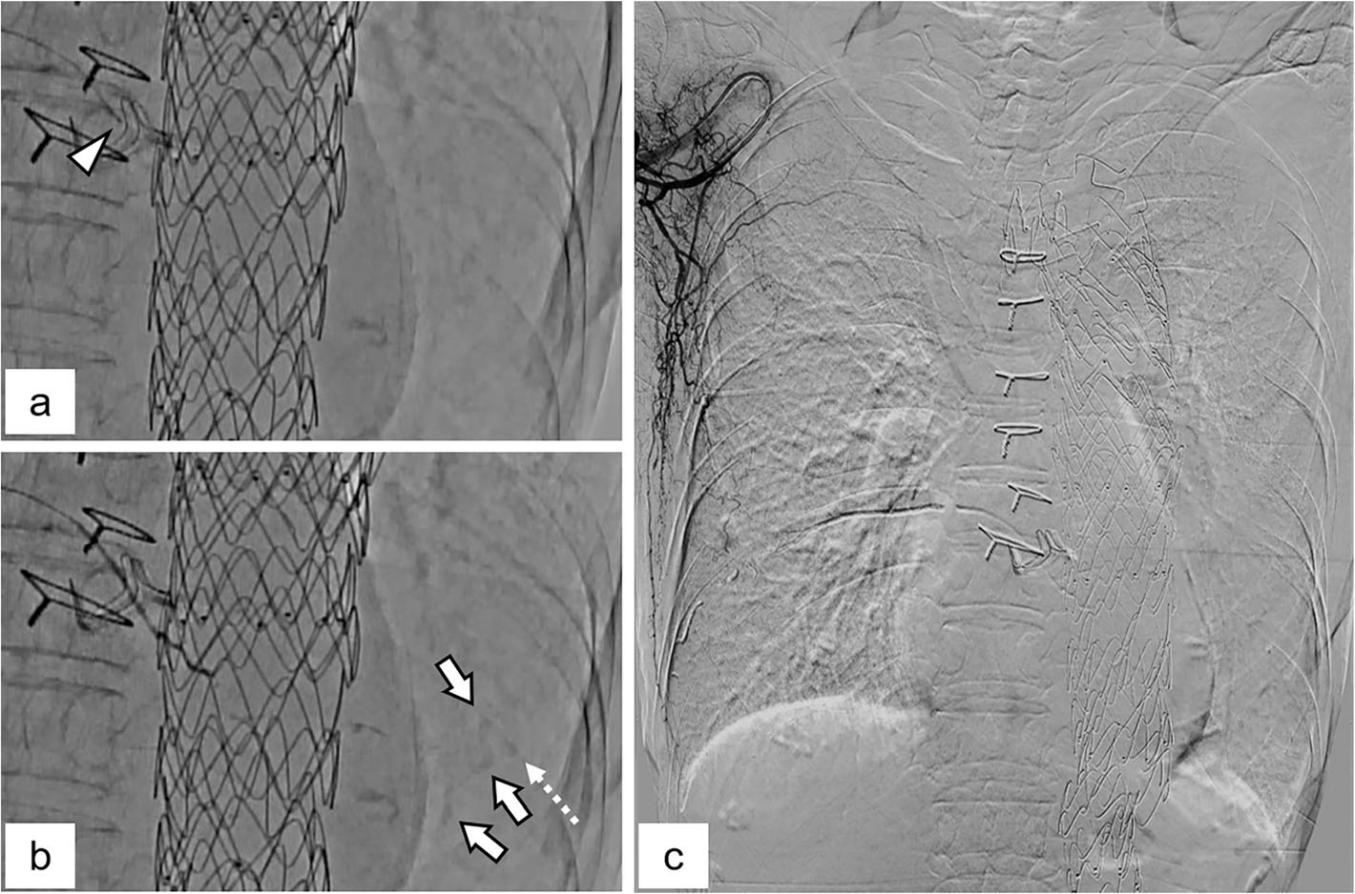
Transcatheter arterial embolization (TAE). **A** Pre-TAE image. The arrowhead indicates the tip of the microcatheter placed at the proximal side of the left pulmonary ligament artery (PLA). **B** Angiographic image acquired during TAE. The depositions of the Lipiodol at the sites corresponding to the pseudoaneurysm (dashed arrow) and abnormal vessels (arrows) are visible. **C** Digital subtraction angiography of the right subscapular artery after TAE. The pseudoaneurysm and abnormal vessels of the left PLA disappeared

An 81-year-old woman was transferred to our hospital by ambulance with massive hemoptysis. She reported a gradually increasing frequency of blood sputum for one month prior to this episode. The patient had a history of aortic valve replacement combined with graft replacement of the ascending aorta and aortic arch and graft replacement of the thoraco-abdominal aorta for dissecting aortic aneurysms 5 and 4 years prior, respectively. She also underwent thoracic endovascular aortic repair (TEVAR) for impending rupture of a descending aortic aneurysm 2 years prior. The patient was taking aspirin (100 mg/day). On admission, she was conscious and alert and her vital signs were stable. Her blood oxygen saturation level was 93% on room air. A complete blood count analysis revealed a hemoglobin level of 10.0 g/dL, white blood cell count of 8,500/µL, and platelet count of 144,000/µL. Serum biochemical tests showed no obvious abnormalities except for a slightly elevated C-reactive protein level (0.8 mg/dL). No abnormalities were detected on a clotting test. Computed tomography (CT) showed a large atelectasis of the left lower lung lobe adjacent to the descending aorta treated with TEVAR. Unenhanced CT showed a heterogeneous high attenuation area within the atelectasis suggestive of a concomitant hematoma. Contrast-enhanced CT revealed a pseudoaneurysm, and abnormal proliferative vessels with continuity with hypertrophied left pulmonary ligament artery (PLA) were detected within the atelectasis (Fig. 1A and B). Furthermore, the PLA continued to the right subscapular artery via a complex collateral pathway including the right intercostal artery (ICA) (Fig. 1C). Since disruption of the pseudoaneurysm and abnormal vessels of the left PLA were considered the cause of massive hemoptysis, we decided to perform transcatheter arterial embolization (TAE) with the patient’s consent. First, the right femoral artery was punctured and an aortography was performed; however, it did not show any type 1, 2, 3, or 4 endoleaks. Next, an access was obtained via the right brachial artery using a 4-Fr sheath. Diagnostic digital subtraction angiography (DSA) of the right subclavian and subscapular arteries revealed a hypertrophied left PLA adjacent to the stent graft of the descending aorta that originated from the right subscapular artery via a complex collateral blood pathway including the right ICA and thoracodorsal artery (Fig. 2A). We subsequently attempted to select the left PLA with the triple coaxial (triaxial) system, which consists of a 155-cm-long 1.9-Fr microcatheter (Carnelian MARVEL, Tokai Medical Products, Kasugai, Japan), a 110-cm-long 2.7-Fr high-flow microcatheter (Sniper 2 High-Flow; Terumo, Tokyo, Japan), and a 65-cm-long 4-Fr angiographic catheter (Impress; Merit Medical Japan, Tokyo, Japan) and finally reached the proximal side of the left PLA. During the procedure, we confirmed no involvement of the anterior spinal artery by DSA of the intercostal artery. Selective DSA of the left PLA showed proliferation of small tortuous vessels and a pseudoaneurysm at the peripheral side (Fig. 2B and C). In addition, small systemic-pulmonary artery shunts were observed at the peripheral side of this lesion (Fig. 2B). Based on the angiographic findings, we decided to use an *N*-butyl cyanoacrylate (NBCA) (B. Braun, Melsungen, Germany)-Lipiodol (Lipiodol Ultra-Fluid; Guerbet, Roissy, France) mixture for embolization, which should reach the embolic material on the peripheral side of the PLA while avoiding large amounts of embolic material migration to the pulmonary artery branches via the systemic-pulmonary artery shunt. We carefully injected 0.8 mL of an NBCA:Lipiodol (1:6) mixture in a stepwise fashion under fluoroscopic guidance until it reached the pseudoaneurysm and abnormal proliferative vessels (Fig. 3A and B). DSA of the right subscapular artery after TAE showed disappearance of the pseudoaneurysm and abnormal vessels of the left PLA (Fig. 3C). We subsequently performed DSA of the bilateral subclavian arteries and celiac artery and confirmed that none supplied the target lesions. Pulmonary angiography of the left pulmonary trunk performed via the right femoral vein showed no pseudoaneurysm or contrast filling defect due to the systemic-pulmonary artery shunt. After TAE, the hemoptysis was controlled without complications, and the patient was discharged 5 days after TAE. Chest radiography performed 2 weeks after the TAE revealed improved consolidation in the left lower field. Six months after TAE, no recurrence of the hemoptysis was noted.

## Discussion

Hemoptysis is a rare complication of TAA repair that usually occurs after ABF formation. A previous study reported that the incidence of ABF after TEVAR was 0.6% (Czerny et al. 2015a, b) and that endoleak is one of its major causes (Czerny et al. 2015a, b; Karube et al. 2019a, b; Sueyoshi et al. 2018a, b). In addition to ABF, pulmonary artery pseudoaneurysm rupture associated with a mycotic TAA and chronic inflammatory reactions of the lung parenchyma secondary to the dispersion of atheroma and/or cholesterol embolization of the pulmonary vessel caused during a previous TAA rupture have been reported as etiologies of hemoptysis after the TAA repair Ishikawa et al. 2019a, b, 2020a, b; Motohashi et al. 2020a, b).

In the present case, we determined that disruption of the pseudoaneurysm and abnormal proliferative vessels of the left PLA caused the massive hemoptysis based on the CT and angiography findings. Although the precise cause of these changes is unclear, the chronic inflammation change of lung parenchyma secondary to chronic compression from TAA and/or protrusion of atherosclerotic materials into the lung parenchyma during previous impending rupture of TAA, similar to previous cases mentioned above, may have contributed to the development of this condition.

The treatment strategy for massive hemoptysis depends on the etiology and the patient’s condition. In recent years, TAE has been used as a first-line therapy because it is less invasive (Chun et al. 2010a, b). However, in cases after surgical or endovascular repair of the thoracic aorta, its use may be difficult due to difficulty directly approaching the responsible artery. In our case, the preoperative contrast-enhanced CT clearly depicted the continuity of the right subscapular artery–thoracodorsal artery–right intercostal artery–left PLA, which was useful for a preoperative evaluation. We also used a triple coaxial system, which allowed us to traverse highly tortuous collateral vessels. The key to achieving hemostasis in our case was complete occlusion of the pseudoaneurysm and abnormal vessels. Since embolization with metallic coils or particles may result in incomplete treatment due to proximal embolization, we used a low-concentration NBCA-Lipiodol mixture. However, when embolizing with low-concentration NBCA for lesions with shunting between target arteries and pulmonary vessels or involving the ICAs, it is important to pay attention to unintentional embolization of non-target vessels to avoid serious complications such as spinal paralysis and massive pulmonary artery embolism. Balloon-occluded injection of embolic material is considered a useful technique for avoiding migration to non-target vessels (Ye and Zhang 2018a, b); however, in our case, this method was not feasible because the target vessel was distal to a complex collateral pathway that the balloon catheter could not reach.

Other treatment options in our case were retrograde embolization from the pulmonary artery side and direct pseudoaneurysm puncture (Shin et al. 2010a, b). Although retrograde embolization from the pulmonary artery side is theoretically feasible, the target pseudoaneurysm is not always delineated because the pressure of the systemic arteries is markedly higher than that of the pulmonary arteries. Moreover, in our case, abnormal proliferative vessels of the PLA in addition to pseudoaneurysm were considered involved in the hemoptysis; however, retrograde embolization alone could not achieve complete embolization of these abnormal vessels. Direct puncture was considered difficult in the present lesion because the target vessel was small and considered difficult to depict on ultrasonography or non-contrast CT.

## Conclusions

The current case demonstrated that TAE via a complex collateral pathway is a feasible and effective treatment for massive hemoptysis, even after TAA repair.

## Data Availability

The datasets used and/or analyzed during the current study are available from the corresponding author upon reasonable request.

## Abbreviations

TAA: Thoracic aortic aneurysm
TEVAR: Thoracic endovascular aortic repair
CT: Computed tomography
PLA: Pulmonary ligament artery
TAE: Transcatheter arterial embolization
ABF: Aorto-bronchial fistula
ICA: Intercostal artery
DSA: Digital subtraction angiography
NBCA: *N*-butyl cyanoacrylate
